# Palliative care services for cancer patients in Nepal, a lower-middle-income country

**DOI:** 10.1177/26323524211021105

**Published:** 2021-06-08

**Authors:** Deepa Gautam, Sudhir Adhikari

**Affiliations:** Department of Radiation Oncology, B.P. Koirala Memorial Cancer Hospital, Bharatpur 44207, Nepal; Department of Paediatrics, Chitwan Medical College, Bharatpur, Nepal

**Keywords:** cancer, cancer care, end-of-life care, home-based care, hospice, low- and middle-income country, Nepal, pain management, palliative care, terminal illness

## Abstract

With the rise in cancer burden, need for palliative care services has increased simultaneously and majority of people requiring services are from low- and middle-income countries where palliative care is in primitive stage. Nepal is also facing similar challenges of dealing with cancer care and end-of-life care. From its initiation in the early 1990s, there has been gradual progress in the development of palliative care with joint effort of government as well as non-governmental organizations. Morphine, a major milestone for pain management, is being manufactured in the country for nearly a decade, yet morphine equivalence mg per capita is far below the global average. Currently, Nepal has been placed under ‘Category 3a’ with isolated care provision and there are a lot of challenges to overcome to improve the existing services. Majority of hospice and palliative care centres are located in the capital city and only a few in the periphery. Scarcity of treatment centres and expertise, limited finances, lack of awareness among patients and health care workers, and difficult terrain are major barriers for optimal care. Proper implementation of national guidelines, human resource development and integration of palliative care to primary healthcare level would be crucial steps for further improvement.

## Background

Burden of non-communicable diseases including cancer is rising every year worldwide and this has brought further constraint in the developing countries with limited health resources.^1^ Globally, cancer accounts for nearly 10 million deaths per year with annual incidence of about 18 million and 5–year prevalence of 43.8 million.^2^ A significant proportion of patients present in advanced stage of disease where curing the illness seems deemed possible, and these patients form a group that are in utmost need of a palliative care that will alleviate their suffering and ensure better quality of life(QOL). Reports from several low-income countries suggest that almost 80% of end stage cancer patients experience moderate to severe pain lasting on an average for 90 days.^3^ At the same time, there is a need to address the stress and emotional issues that their family and caregivers go through during such a grave situation, and palliative care has emerged as an indispensable part of health care to tackle these issues.

Palliative care is a holistic approach that encompasses care of patients with distressing incurable diseases, managing their pain and other symptoms, providing end-of-life care along with the support to their families and caretakers. World Health Organization (WHO) defines palliative care as

an approach that improves the quality of life of patients and their families facing the problem associated with life-threatening illness, through the prevention and relief of suffering by means of early identification and impeccable assessment and treatment of pain and other problems, physical, psychosocial and spiritual.^4^

Palliative care and pain relief is an essential component of Universal Health Care.^5^

There are about 40 million people globally who require palliative care and 78% live in low- and middle-income countries where palliative care is in primitive stage.^6^ Only 30 countries around the world have advanced palliative care facilities which provide services to 14% of the world population eligible for support at the end of life.^7^ Nepal, being one of the developing countries, is also facing similar problems associated with palliative care and hence, there is an urgent need to upgrade the services to address the sufferings of people in a systematic way.

## Cancer burden in Nepal

In addition to the existing infectious diseases, the incidence of non-communicable diseases including cancer is increasing every year. Two thirds of the total annual mortality is attributable to non-communicable diseases and cancer deaths account for 9%.^8^ Cancer is one of the dreadful chronic conditions which results in significant burden to patients, their families and healthcare system. In Nepal, in the year 2018, there were 26,184 new cases along with 19,413 deaths. The prevalence of cancer is 43,816 cases which is likely an underestimate because most of the cases remain undiagnosed.^2^

## Development of palliative care

Worldwide, focus towards modern palliative care in the form of pain management and end-of-life care needs of the patients with advanced stage cancer was initiated in the late 1960s and about a decade later, it expanded to include the psychosocial and the spiritual care as well. Thereafter, in the late 1980s, it grew as a separate subspecialty of general medicine.^9^

A palliative care model for resource-poor settings has been demonstrated in Kerala, a state of India, provides a model for Nepal, demonstrating that quality services is possible with active participation of trained community volunteers and family. This model uses local resources to provide home-based, community-based and outpatient clinics-based care.^10^ Non-governmental organizations operated free palliative care service model can be useful as well for running similar development projects in other developing countries.^11^

Palliative care services for cancer patients, in Nepal, began in 1991 when the department of oncology was started at Bir Hospital, Kathmandu^12,13^ The department later expanded to involve a medical oncology team with expertise in palliative care which now provides clinical services as well as academic training for post-graduate medical students.^14^ In 1992, the first cancer hospital in Nepal, B.P. Koirala Memorial Cancer Hospital (BPKMCH), Bharatpur, was established where cancer patients could receive palliative care services along with the curative therapy.^15^ A dedicated palliative care unit with all forms of inpatient, outpatient and home-visit services as well as a separate hospice facility has been providing services to the needy patients since then. Various public and private hospitals are also providing palliative care services in different parts of the country. The government of Nepal, Public Health Service Act 2018 has also included palliative services in the definition of ‘health service’ providing legal framework towards palliative care development.^16^

Non-governmental organizations (NGOs) have played key role in providing services as well as running training programmes in the country including International Nepal Fellowship Nepal in Green Pastures Hospital, Pokhara is providing palliative care services since 2009 in the under-resourced Western Region.^17^ Similarly, United Mission to Nepal supports palliative care development by establishing hospitals in rural parts of the country to provide holistic care and International Network for Cancer Treatment and Research (INCTR) has been involved in providing education and training as well as financial support to the hospices.^17,18^

On the basis of various dimensions of palliative care, Nepal is classified as ‘Category 3a’ with isolated palliative care provision.^7^ This means that the development of palliative care services are still patchy in scope and not well supported, heavily donor dependent funding, limited morphine availability, and services not proportionate to the huge population size.^7^

The American Society of Clinical Oncology (ASCO), after recognizing the limitations and challenges that a resource-poor country has to face regarding the implementation of palliative care, has suggested consensus guidelines for resource-constrained settings to integrate palliative care into standard oncology care. The four suggested models are basic (Primary health care), limited (District), enhanced (Regional), maximal (National) based on staffing requirements, roles and training needs of team members, psychosocial support, spiritual care, and opioid analgesics.^19^ In resource-constrained settings, basic palliative care can be provided by the staff in an institute especially doctors, nurses and volunteers, after they are given adequate training. In Nepal, in those centres where there is no separate palliative care unit, public hospitals, private hospitals, medical colleges, for instance, the attending physicians often oncologists, provide palliative care services. Apart from oncologists, internal medicine physicians, anaesthetists, general practitioners can also be trained and involved in providing services.

## Education and training

The Nepalese Association of Palliative Care (NAPCare), a non-profit non-governmental organization was established in 2009, to improve palliative care services in the country and to create awareness and educate the healthcare personnel about palliative care.^20^ NAPCare has developed the National Strategy for Palliative Care, which focuses on pain management in co-ordination with Ministry of Health (MoH) and WHO with support from Two Worlds Cancer Collaboration, Canada.^17,20,21^

With the joint effort of national and international organizations, palliative care training programmes have been run in Nepal since 2009.^13^ Network for Cancer Treatment and Research (NNCTR), a non-profit non-governmental organization, has been working in the field of cancer care since 2000. To date, 2214 medical, nursing and public health students have been sensitized to palliative care with one-day course and 7 doctors and 20 nurses have received short-term palliative care training of one month.^22^ With the joint collaboration of the NNCTR, NAPCare and BPKMCH, one-month palliative care training programme for doctors and nurses has been conducted in 2010 and 2012.^23^ NAPCare and National Health Training Centre under MoH have jointly started two-week training twice a year since 2013 but in recent years, the duration has been reduced to six days.^13^ Similarly, BPKMCH had conducted two six-week courses in palliative care nursing for health professionals from different parts of the country. Hospice Nepal has also trained 48 physicians and nurses from various institutions by running two 6-week courses on palliative care.^18^ BPKMCH and Hospice Nepal have separately conducted two-day sensitizing courses for health workers as well.

The National Academy of Medical Sciences, a Bir Hospital based teaching institution in Kathmandu, has also been training its post graduate medical trainees (residents and fellows) about palliative care.^13^ However, with regards to undergraduate education, although the WHO has emphasized for compulsory palliative care education and it has been included in undergraduate medical curriculum in various countries, in Nepal, two medical institutions, Patan Academy of Health Science and Institute of Medicine have only introduced the topic in the curriculum for undergraduate students for just over a decade. A study conducted in one these institutes had demonstrated inadequate knowledge and perception of palliative care among undergraduate medical students.^24^

## Palliative care services in Nepal

Hospice Nepal is the first formally established modern hospice centre in Nepal which was started in the year 2000 in Kathmandu Valley.^18,25^ Currently, there are five hospices dedicated to caring cancer patients and four palliative care units of major cancer hospitals of the country. (Table 1) Maiti Nepal which runs Sattighatta Hospice and Sonja Kill Memorial Hospice, Nava Kiran Plus, Blue Diamond Society are other key organizations which provide care to patients with other life-limiting illnesses like HIV/AIDS, spinal injuries, human-trafficking survivors.^13,26^ The majority of them are located in the capital city of the country, and only a few are located outside Kathmandu Valley. There are few hospice centres which blend spiritual and religious aspect of caring patients with the medical care such as, the Pashupatinath Hospice, located in the vicinity of one of the most famous religious places in Nepal, the Pashupatinath Temple. There is also a hospice in the Shechen Monastery at Bouddha established by a Buddhist charitable foundation named Karuna-Shechen which provides palliative care to needy patients.^17,23^

**Table 1.** Hospices and hospitals with palliative care units in Nepal.

	Year established	Location	Inpatient palliative care beds	Home care services	Website link
Hospices					
Pashupatinath Hospice	1995	Kathmandu	–	–	https://www.facebook.com/people/Pashupatinath-Hospice/100009488305083
Hospice Nepal	2000	Lalitpur, (Kathmandu Valley)	9	Yes	http://www.hospicenepal.org.np/page/how-it-all-began
Karuna-Shechen Hospice	2004	Kathmandu	–	–	https://karuna-shechen.org/
BPKMCH Hospice	2004	Bharatpur, Chitwan	15	Yes	https://bpkmch.org.np/departments/hospice.php
Thankot Hospice Centre	2007	Kathmandu	10	Yes	https://thankot-hospice-centre.business.site/
Hospitals with palliative care units					
Bhaktapur Cancer Hospital	2004	Bhaktapur (Kathmandu Valley	9	Yes	http://www.bhaktapurcancerhospital.org/
BPKMCH	2004	Bharatpur, Chitwan	450 bedded hospital separate beds not allocated	Yes	https://bpkmch.org.np/departments/hospice.php
The Binaytara Foundation Cancer Centre–Hospice & Palliative Care Programme	2016	Kathmandu	–	Yes	https://cancer.binayfoundation.org/
	2018	Janakpurdham	25 bedded hospital (separate beds not allocated)	Yes	

BPKMCH, B.P. Koirala Memorial Cancer Hospital.

In Nepal, home-based care for terminally ill cancer patients is being provided by health workers including those working with the Binaytara Foundation Cancer Centre–Hospice & Palliative Care Programme. This programme has focused on cost-effective home-based care initially in Kathmandu Valley and later expanding to Janakpurdham, a city in southern Nepal.^27^

## Opioid use and pain management

To live a pain-free life is the right of cancer patients,^28^ and thus, opioid analgesics are vital for symptom palliation. The WHO analgesic ladder has been a simple and effective guide for over three decades towards reducing the morbidity due to pain in cancer patients. Among the medications used, opioids have played the most significant role in controlling moderate to severe pain in those patients.^29^ Morphine, the most commonly used opioid as well as the initial drug of choice in managing the severe cancer-induced pain, had been a major breakthrough in the development of palliative care. According to WHO, immediate-release oral morphine must be available and accessible to all patients requiring it and slow-release formulations should be made available as well.^29^

A Nepalese pharmaceutical company was licenced to manufacture morphine in 2009, and 10-mg immediate-release tablets manufacturing started in 2011 and followed by sustained-release oral morphine and syrup.^23^ Department of drug administration under ministry of health and population regulates the sales and distribution of opioids and other narcotic drugs.^30^ In 2015, it was found that Nepal had morphine equivalence (ME) of 0.27 mg per capita as compared to the global average of 61.5 mg per capita and South-East Asia Region of 1.7 mg per capita.^31^ Despite the strong emphasis on morphine by the WHO, there are instances of inconsistencies in its availability in the country because of the limited production and supply not being able to meet the demand^14^ in contrast to the scenario of the developed world where epidemics of opioid overdose exist.^32,33^

## Financial aspects of palliative care

Nepal has recently been upgraded from low-income to lower-middle-income economy with per capita income of US dollars ($) 1,090.^34^ The government of Nepal provides treatment support equivalent to about US$855 for cancer patients and recently, Nepalese government has introduced health insurance system through ‘Health Insurance Board (HIB)’ which provides additional support for the treatment of insured citizens.^14,35^ This also covers daily expenses of Nepalese Rupees (NRS) 500 (US $ 4.28) per day for patients receiving palliative care. Although this looks a bit encouraging for Nepalese patients with financial limitations, cancer management remains costly and this amount becomes insignificant compared to the cost of treatment. Apart from the treatment cost, expenses for travelling hundreds of miles, food, lodging for patients and their caretakers becomes a burden, and on top of that, loss of daily income of both patients and their caretakers. Many patients are compelled to sell their property, livestock, jewelleries and any other assets or take loans to pay for treatment and end up in debt.^36^ The financial assistance by the government is provided once the diagnosis is established and only available at treatment centres that are designated to provide this support. Hence, out-of-pocket expenditure remains the primary source of funding. In such a scenario, early palliative care referral for those not benefitting from expensive curative treatment can prove to be a cost effective approach for both patients and the government.^37^

## Barriers to effective palliative care services

The potential limiting factors for implementation of successful palliative care are provider-related, patient-related and healthsystem-related. Limited services with a lack of infrastructure and skilled human resources, inconsistent drug availability as well as improper regional and national strategy and guidelines are hindering the path of palliative care development in Nepal.^13,38^ Healthcare providers, at times, are reluctant to refer the patients for palliative care being unaware of its benefits or fear of losing the continuity of treatment and follow-up of the patients with them after seeking treatment elsewhere.^38^

Lack of awareness by patients and their families about the improved quality of life a distressed patient can live with the help of palliative care leads to opting for alternative treatments. There are instances when after knowing that disease is incurable or in advanced stage, instead of taking palliative care, they either stop their treatment and go back home, or visit some quacks who promise them to cure the disease but instead cause further damage their financial as well as health conditions. It is not uncommon that the patient does not know his or her diagnosis and prognosis because family members are reluctant to disclose the diagnosis and prognosis to the patient fearing that he or she will lose hope to live.^39^

Clear communication with patients and their families about the disease, treatment and prognosis has been a major limitation that is observed among the service providers in Nepal and the major reason being the lack of expertise.^14^ There is hardly any professional counsellor even in a tertiary cancer centre which leaves this task to doctors or nurses who do not have adequate time for proper counselling, and lack skills and training in this area.

In Nepal, 83% of the total area is hilly and mountainous; this difficult terrain acts as a challenge for accessibility of overall health services including palliative care. Thus palliative care must be integrated in the community health level to reach everyone who needs it. Mid-level health workers in government services such as health assistants, auxiliary health workers who are the major service providers in the rural areas of the country are found to be enthusiastic in learning about palliative care and providing care in the community.^40^

Also, a Female Community Health Volunteers (FCHVs) programme, which was started in 1988, acts as a strong linkage between the health system and the community. These volunteers are primarily involved in maternal and child health services programmes and hence, supporting the implementation of the community-based health interventions.^41^ Their involvement in basic palliative care could also be an important step in the path of universal coverage.

## Socio-cultural and spiritual aspects

Nepal is a multicultural, multi-ethnic, religiously secular country with predominantly Hindu and Buddhist populations, and each culture has its own norms and values, rituals during life and death.^14,17^ Most people wish to be at home with their family and relatives during their last days of life, and support at home and community level is of utmost importance. Despite socio-cultural and spiritual issues being an important part of palliative care, these are often ignored by both the service providers and the patients and their families. Spiritual health interventions for cancer patients create a positive faith and peace of mind which gives strength to face the illness and gives the sense of symptoms being improved and thus, improving the quality of life.^42^

Bereavement care is another crucial aspect which is not optimal even in the developed countries^43^ and almost non-existent in our country. Coping with the devastating situation of losing loved ones is extremely difficult for the family members, and supportive services from trained healthcare providers are helpful for gaining strength and support. In Nepal, friends and relatives visit the bereaved families for console and express their condolences till last rites are over, usually 13-day period, after which they do not continue their bereavement support to those families.

## Paediatric palliative care

Although WHO and American Academy of Paediatrics has recommended the initiation of palliative care at diagnosis of childhood malignancy, often there are delays in the discussion about the care with the family and start of the palliative care.^44^ Lack of dedicated centres, competent service providers, delayed referral, and effective communication between health service providers and the patients and the families are the common reasons for such delays.^45^

Providing optimal relief of bothersome symptoms including pain that children suffer is an important aspect of the paediatric palliative care. Identifying and managing the distressing symptoms, and providing a holistic care requires interdisciplinary collaborations among paediatric oncologists, paediatric palliative care experts, psychiatrists, psychologists and child life specialists.^46^

Discussion about the health status of children to their parents or guardians is a sensitive issue. A compassionate and transparent communication about the prognosis of the disease, treatment plan and end-of-life care, keeping in mind the psychosocial impact that it has to the patient and the caretakers and thus developing patient-centred and family-centred goals is a crucial part of paediatric palliative care.^44,46^ Hence, trainings and mentorship programmes for care providers are essential for developing excellent communication skills to conveying the correct information effectively to the patients and the families.^45^

In Nepal, Kanti Children’s Hospital, Kathmandu, BPKMCH, Bharatpur and Bhaktapur Cancer Hospital, Bhaktapur are the three major centres that are treating the paediatric cancers as well as providing palliative care services to the children in need.^18,47^

## Future needs and recommendations

- Complete implementation of national guidelines in palliative care would be beneficial for the service providers to give optimal uniform services throughout the country.
- Inclusion of palliative care in the curriculum of undergraduate and postgraduate medical and paramedical courses to sensitize the students to the subject.
- Integration of palliative care to the primary healthcare level with mobilization of local human resources such as community volunteers to optimize the accessibility.
- Establishing separate palliative care units at the tertiary hospitals and as well as regional and district levels.
- Opening of hospice centres in every region of the country.
- Prioritizing cost effective home-based care.

## Conclusion

The increasing cancer burden in Nepal has led to the rise in patients requiring palliative care. For last three decades, Nepal has been progressing in providing services to the terminally ill cancer patients, and the local opioid production has been a milestone in the path of development of palliative care. NAPCare has assisted the government of Nepal to formulate national guidelines, yet there are still significant gaps in its implementation.

National and International Non-governmental organizations have played a significant role in expanding palliative care in the country. Despite the assistance provided by the government, financial issue still remains a major challenge for the most patients and their families. Effective communication with patients and the family members is crucial for providing optimal services. Accessible, affordable and socially acceptable quality palliative care at community level should be the main goal for universal coverage of palliative care.
